# Letter from Sir John Sinclair, Bart. to the President of the College of Physicians, at Edinburgh, on the Remedy Lately Discovered for Calculous Complaints

**Published:** 1812-07

**Authors:** John Sinclair

**Affiliations:** 27, Old Burlington-street


*For the Medical and Physical Journal.*

LETTER *from* SIR JOHN SINCLAIR, BART. *to the* PRESIDENT *of the* COLLEGE *of* PHYSICIANS, *at* EDINBURGH, *on the* REMEDY *lately discovered for* CALCULOUS COMPLAINTS.

SIR,

I AM persuaded that you will concur with me in opinion, that there are no complaints incident to mankind, for which it is more desirable to discover a cure, or even a safe and effectual means of preventing, than those of a calculous nature. The miseries felt by those who are afflicted with the stone or gravel, are dreadful beyond description; and any cure effected by surgical operation, is not only of a nature peculiarly severe, and often fatal, but does not always prevent a repetition of the complaint. It is with much pleasure, therefore, that I call the attention of the respectable body over whom you preside, to the means, which have been recently discovered, by which calculous complaints may, there is every reason to hope, be prevented from proving, in future, so formidable a source of human misery.

The paper printed in the Philosophical Transactions, An. 1810, entitled "Observations on the Effects of Magnesia in preventing an increased formation of the Uric Acid," written by Mr. William T. Brande, is the first suggestion, so far as my information reaches, that has been published, recommending magnesia as an antidote to the formation of stone or gravel.

The inquiries of that eminent surgeon, Mr. Home, into the functions of the stomach, and his discovery that liquids pass from the cardiac portion into the circulation of the

---

blood, blood, (Philos. Trans. April 1808,) led him to consider that the generality of calculous complaints might possibly be prevented, by introducing into the stomach such substances as are capable of preventing the formation of uric acid; and that this mode of treatment would have many advantages over the usual method, which consists in attempting to dissolve the uric acid after it is formed.

He consulted, it would appear, Mr. Hatchett, on the substance most likely to produce this effect; and that respectable chemist concurred with him in opinion, that magnesia, from its insolubility in water, was peculiarly well adapted for the purpose, as it would remain in the stomach, until it should combine with any acid, or be carried along with the food to the pylorus.

These gentlemen, anxious to have a complete investigation of a subject so peculiarly interesting, requested Mr. Brande to assist them in the prosecution of it; and many opportunities having occurred of carrying on the inquiry, during an attendance on patients afflicted with calculous complaints, the results of their joint labors were communicated to the Royal Society in the important paper I have alluded to, which is earnestly recommended to the consideration of every medical person, who may have patients afflicted with such complaints under his care.

As calculous matters are not uniformly of the same description, it is certainly desirable to ascertain, by the assistance of some medical person, the specific nature of the complaint with which any individual is afflicted; but, being anxious that those who are at a distance from medical aid, or who cannot, perhaps, afford to procure it, should be able to avail themselves of this new discovery, I took the liberty of applying to Mr. Brande for a short statement of the manner in which the remedy ought in general to be applied; and he, with that liberality which belongs to true genius, has furnished me with the following particulars.

"The best method of giving the magnesia is in plain water, or milk, to be taken in the morning early, and at mid-day. If the stomach is weak, and this produces flatulency, or uneasy sensations, some common bitter, such as gentian, may be taken with it: if it purges, a little opium may be added.

"The mode in which magnesia operates in preventing the formation of stone or gravel, Mr. Brande believes to be, by preventing the formation of acid in the stomach.

"Where there is a stone actually formed in the bladder, it is of advantage (if the case is a proper one for the use of magnesia) by preventing the increasing in size.

"The

"The dose of magnesia must intirely depend upon the circumstances of the case—*generally*, five grains, twice or thrice a day to children below ten years of age; fifteen and twenty grains to adults.

"Mr. Brande has always given common magnesia. The calcined may occasionally be used with advantage. Any omission of the remedy for a time must entirely depend on the state of the patient.

"In regard to diet, no particular regimen is necessary. Animal food may be taken in the same quantities, and of the same sorts as usual. The patient should avoid strong wines, such as Port and Madeira, and indeed should abstain from pure wine altogether. Spirits should not be taken in any form. Lemons and vinegar need not be absolutely avoided, unless the bowels are too open. Oranges and other fruits, when ripe, may be taken."

There is every reason to hope, from the success that has already attended the application of this remedy in various cases, that it may prove an effectual means of preventing the greatest proportion of calculous complaints; and it is impossible to foresee, to what a state of improvement it may yet be brought, in the course of further experience, when the attention of a number of able and learned men (as I trust will be the case) is directed to its application.

Perhaps there is no disorder to which mankind is liable, for which nature has not provided some remedy, or at least some means of alleviation, though, in both respects, much remains to be learnt; and any discovery that can be serviceable in calculous complaints, from the infinite distress they occasion, must prove, in a peculiar manner, gratifying to every real friend to the happiness of the human race.

I have the honor to be, Sir,

Your humble and obedient Servant,

27, *Old Burlington-street*, JOHN SINCLAIR.

*May* 7, 1812.

P.S. Since this letter was written, I have met with another interesting paper, containing some valuable hints regarding the Cure of Gravel.* Instead of soda-water, or the *aqua mephitica alkalina*, the author recommends, for adults, the following medicine: "a drachm, or two scruples, of mild vegetable alkali, otherwise called salt of wormwood, or salt

* Entitled, "On the Effects of large Doses of mild vegetable Alkali, or Potassa Carbonata, in Gravel, and the beneficial Effects of Opium combined with it." By Gilbert Blane, M.D. Physician to the Prince of Wales.

of of tartar, dissolved in two ounces of water, sweetened with two drachms of honey, to be taken with half an ounce of lemon-juice three times a-day." On this medicine it is to be observed—1. That honey is serviceable in calculous complaints, and is preferable to sugar. 2. That the use of honey renders any opening medicine in general unnecessary, even when opium, as is subsequently recommended, is administered. 3. That, according to the above plan, more alkali is given than the stomach could otherwise bear. 4. That though the virtue of a large proportion of the alkali is probably impaired by the addition of the acid, yet that this is more than compensated by its accommodating the stomach to large doses of the alkali. 5. That the mischievous effects on the stomach, which so often take place from the use of the alkalies alone, are thereby prevented.

Where there is much pain combined with inflammation, bleeding may be necessary, or the addition of nitre to the saline draught; but where there is much pain, without the suspicion of inflammation, opium is highly advisable, in the proportion of from five to ten drops of laudanum, to each dose of alkali. The cure is thus rendered more expeditious, more certain, and more permanent. Indeed one of the principal objects of this tract alluded to, is to inculcate, from the author's experience, that the addition of opium gives greater certainty and permanency to the cure.

It may be further remarked, that, as there is a great variety in cases and constitutions, and as it appears by chemical analysis, that calculous concretions vary in the nature of their component parts, so must the remedies, according to these diversities. There are few cases, however, in which either this or the preceding plan of cure may not be successfully applied.

